# Identification and Nearly Full-Length Genome Characterization of Novel Porcine Bocaviruses

**DOI:** 10.1371/journal.pone.0013583

**Published:** 2010-10-25

**Authors:** Wei-xia Cheng, Jin-song Li, Can-ping Huang, Dong-ping Yao, Na Liu, Shu-xian Cui, Yu Jin, Zhao-jun Duan

**Affiliations:** 1 State Key Laboratory for Molecular Virology and Genetic Engineering, National Institute for Viral Disease Control and Prevention, China Center for Disease Control and Prevention, Beijing, People's Republic of China; 2 School of Basic Medical Sciences, Lanzhou University, Lanzhou, People's Republic of China; 3 Medical School of Nanjing University, Nanjing, People's Republic of China; University of Hong Kong, Hong Kong

## Abstract

The genus *bocavirus* includes bovine parvovirus (BPV), minute virus of canines (MVC), and a group of human bocaviruses (HBoV1-4). Using sequence-independent single primer amplification (SISPA), a novel bocavirus group was discovered with high prevalence (12.59%) in piglet stool samples. Two nearly full-length genome sequences were obtained, which were approximately 5,100 nucleotides in length. Multiple alignments revealed that they share 28.7–56.8% DNA sequence identity with other members of Parvovirinae. Phylogenetic analyses indicated their closest neighbors were members of the genus bocavirus. The new viruses had a putative non-structural *NP1* protein, which was unique to bocaviruses. They were provisionally named porcine bocavirus 1 and 2 (PBoV1, PBoV2). PBoV1 and PBoV2 shared 94.2% nucleotide identity in *NS1* gene sequence, suggesting that they represented two different bocavirus species. Two additional samples (6V, 7V) were amplified for 2,407 bp and 2,434 bp products, respectively, including a partial *NP1* gene and the complete *VP1* gene; Phylogenetic analysis indicated that 6Vand 7V grouped with PBoV1 and PBoV2 in the genus of *bocavirus*, but were in the separate clusters. Like other parvoviruses, PBoV1, PBoV2, 6Vand 7V also contained a putative secretory phospholipase A_2_ (sPLA_2_) motif in the *VP1* unique region, with a conserved HDXXY motif in the catalytic center. The conserved motif YXGXF of the Ca^2+^-binding loop of sPLA2 identified in human bocavirus was also found in porcine bocavirus, which differs from the YXGXG motif carried by most other parvoviruses. The observation of PBoV and potentially other new *bocavirus* genus members may aid in molecular and functional characterization of the genus bocavirus.

## Introduction

Animals play an important role in the spread of human diseases. Since the development of civilization, animals, especially livestock and fowls, have had a close association with human beings. Previous studies have indicated that 61% of pathogens known to infect humans can be transmitted between humans and animals, and that 75% of emerging pathogens are zoonotic, especially protozoa and viruses [1]. The transmission of bird flu to humans is a typical example. Likewise, members of the family *Parvoviridae* also represent significant pathogens in human and animal diseases [2]–[4]. The family *Parvoviridae* includes two subfamilies, *Parvovirinae* and *Densovirinae*; the former infect vertebrates and the latter infect invertebrates. The subfamily *Parvovirinae* can be divided into six genera: *Parvovirus*, *Erythrovirus*, *Dependovirus*, *Amdovirus*, *Bocavirus*, and a new proposed genus *Hokovirus* [4]–[6].

Members of the genus *Bocavirus* are virions consisting of an isometric, non-enveloped capsid, which is round with icosahedral symmetry. The bocavirus genome is not segmented and contains a single molecule of linear, positive- or negative-sense, single-stranded DNA of 4,000–6,000 nucleotides in length. Known members of bocavirus include bovine parvovirus (BPV), minute virus of canine (MVC) and Human bocaviruses 1–4. The MVC genome shares about 43% identity with that of BPV at the nucleotide level, with the *NS1*, *VP1*, and *NP1* proteins being 33.6%, 41.4%, and 39% identical to those of BPV, respectively [7], [8]. BPV was first identified in 1961 in samples from calves with diarrhea [9], while MVC was first isolated from canine fecal samples in 1970 [10]. Subsequent studies indicated that these two viruses had close relationships with respiratory and enteric infections in animals, especially in younger animals [4], [7]. In 2005, Allander *et al.* reported the detection of a new human parvovirus in children with acute respiratory tract infections, showing 42–43% amino acid identity to the nearest neighbor MVC and BPV in both major ORFs that encode the *NS1* and *VP1* proteins. It was provisionally categorized into the genus bocavirus and named Human Bocavirus (HBoV) [11]. Recently, HBoV2, HBoV3, and HBoV4 have been discovered and all have been categorized into the genus bocavirus [12], [13]. These human bocaviruses had close relationships with respiratory and enteric infections in humans, but their connection with these infections has not been proven by animal model [11]–[16].

In recent years, with the development of random amplification methodologies and high-throughput sequencing, more new viral pathogens have been identified in samples from humans and animals [17]–[19]. Sequence-independent single primer amplification (SISPA) is a primer-initiated technique by which nucleic acids of an unknown sequence can be amplified with sequence-independent PCR methods using a single primer [20]. Since this method has been used, some new human and animal viruses, including new bovine parvovirus species, bungowannah virus, new torque teno mini virus species, and human bocavirus 1, have been discovered [11], [18], [21], [22]. Using this method, we identified a novel group of viruses in the stools of piglets, the closest neighbors to which are MVC, BPV and HBoV. We provisionally named them porcine bocavirus (PBoV) and report their genomic characterization here.

## Materials and Methods

### Study participants and sample collection

From September to November of 2006, 397 fecal specimens were collected from healthy piglets (<15 days of age) from three different farms and several sporadically distributed families that raise pigs in Lulong County, China. All fecal specimens were frozen at −80°C until further processing.

### Identification of novel viruses

We used SISPA to screen and identify viral agents, according to previous protocols [11], [18], [23]. Ten stool samples, diluted 1∶5 (wt/vol) in phosphate-buffered saline, were mixed together. To remove bacteria and cells present in the feces, the mixed samples were centrifuged (10,000×*g*, 10 min), and the suspensions were then separated through 0.45 µm and 0.22 µm filters (Ultrafree-MC, Millipore). The filtered suspensions were ultracentrifuged (200,000×*g*, Beckman rotor, 180 min) to pellet the viral particles; the pellet was then resuspended in 200 µL of sterile saline solution. Next, 100 units of DNAse I (Promega) and 3 µL RNAse A (Qiagen) were added to the viral resuspension and it was incubated at 37°C for 90 min. Viral DNA was extracted from the stool resuspension using the QIAamp® Viral DNA Mini kit (Qiagen), according to the manufacturer's protocol. The DNA was redissolved in 50 µL of RNase- and DNase-free water and stored at −80°C until further processing.

To detect viral DNA, 20 µL of extracted DNA was mixed with 2 µL of 10 µM primer FR26RV-N (GCCGGAGCTCTGCAGATATCNNNNNNNNNN), 1 µL of 10 mM dNTP, and 2.5 µL of 10×Ecopol buffer (New England Biolabs). The samples were then incubated at 94°C for 3 min and chilled on ice for 2 min; then, 2.5 units (0.5 µL) of 3′-5′exo-Klenow DNA polymerase (New England Biolabs) was added, before incubating at 37°C for 1 h, followed by chilling on ice for 2 min. Then, 2.5 units (0.5 µL) of 3′-5′exo-Klenow DNA polymerase was added, and the reaction was incubated again at 37°C for 1 h, followed by enzyme inactivation at 75°C for 10 min and chilling on ice for 2 min. Next, 5 µL of the above reaction mix was used as a template in the following PCR, mixed with 40 pmol of primer FR20RV (GCCGGAGCTCTGCAGATATC), 2.5 mM MgCl_2_, 1 µL of 10 mM dNTP, 5 µL of 1×GeneAmp PCR buffer II and 0.5 µL of AmpliTaq Gold DNA polymerase (Applied Biosystems) in a 50 µL reaction. After 10 min at 94°C, 40 cycles of amplification (94°C for 1 min, 55°C for 1 min, and 72°C for 2 min) were performed, followed by a final extension at 72°C for 10 min.

PCR products were separated on a 1.5% agarose gel and fragments between 300–1500 bp in length were excised and extracted using the QIAquick Gel Extraction kit (Qiagen). The extracted products were cloned, and the plasmid inserts were sequenced.

Contigs or singlet sequences that failed to assemble were aligned against the NCBI nr database using the BLASTn and BLASTx algorithms. An E-value cutoff of 1×10^−5^ was used according to previous study [23].

### Detection and genomic sequences of porcine bocavirus

According to the sequence obtained from SISPA, two primers (forward: 5′-AAACTGGTTCCTGAGC-3′ and reverse: 5′-CAGTGAAACAGCGTCT-3′) were designed to amplify a 208-nt region within the ORF of *NS1*. After 5 min at 94°C, 35 cycles of amplification (94°C for 45 s, 50°C for 45 s, and 72°C for 1 min) were performed, followed by a final extension for 10 min at 72°C. PCR products were verified by sequencing and submitted to GenBank (Acc. No. HM053674-HM053692).

To obtain complete sequences of PBoV, specific primer PCR and genome walking (kit D316; TaKaRa) were used (all primer sequences are available on request). The positive PCR products were cloned, and the plasmid inserts were sequenced. The nearly full-length sequences, except the termini, were submitted to GenBank (Acc. No. HM053693-HM053694). Two additional sequences (6V and 7V) obtained from this process were also submitted to GenBank (Acc. No. HM053672-HM053673).

### Sequence and phylogenetic analyses

The nucleotide and deduced amino acid sequences of PBoV were compared with entries in the GenBank database. The sequences were aligned and manually adjusted using ClustalW. Phylogenetic trees were determined by the neighbor-joining (NJ) method, implemented in the Molecular Evolutionary Genetics Analysis (MEGA) 4.1 software package. Various nucleotide substitution models were examined and phylogenetic trees of similar topology were generated. A bootstrap resampling (1,000 replicates) was used to assess the reliability of individual nodes for each phylogenetic tree. Recombination analysis between PBoV and other parvoviruses was conducted using SimPlot.

## Results

### Molecular virus screening of porcine stool samples

Two pools of viral resuspension were extracted, amplified and, cloned. In total, 120 clones were sequenced, and the sequences were searched against those in the NCBI databases using the BLASTx and tBLASTx programs. The clones included gene fragments of human, phage, virus, bacteria, and unknown sequences. Of the 120 clones analyzed, 18 were significantly similar to viral sequences (E<1×10^−5^); 16 of these matched porcine kobuvirus, porcine circovirus, and frog virus with high sequence identity, whereas the other two sequences, sequences A and B, were found to be Parvovirus-like sequences. These two sequences did not share similarity with database sequences at the nucleotide level by BLAST searching using default parameters. Nevertheless, the deduced amino acid sequence A matched the *NS1* gene of MVC (40% identity, E = 8×10^−14^) and HBoV2 (44% identity, E = 4×10^−6^) using the tBLASTx algorithm. Likewise, the deduced amino acid sequence B matched the *VP1* gene of MVC (57% identity, E = 6×10^−34^) and HBoV (68% identity, E = 1×10^−28^) using the tBLASTx algorithm. We provisionally indicated the virus as “porcine bocavirus (PBoV).”

### Detection of PBoV in healthy piglets

**Figure 1:**
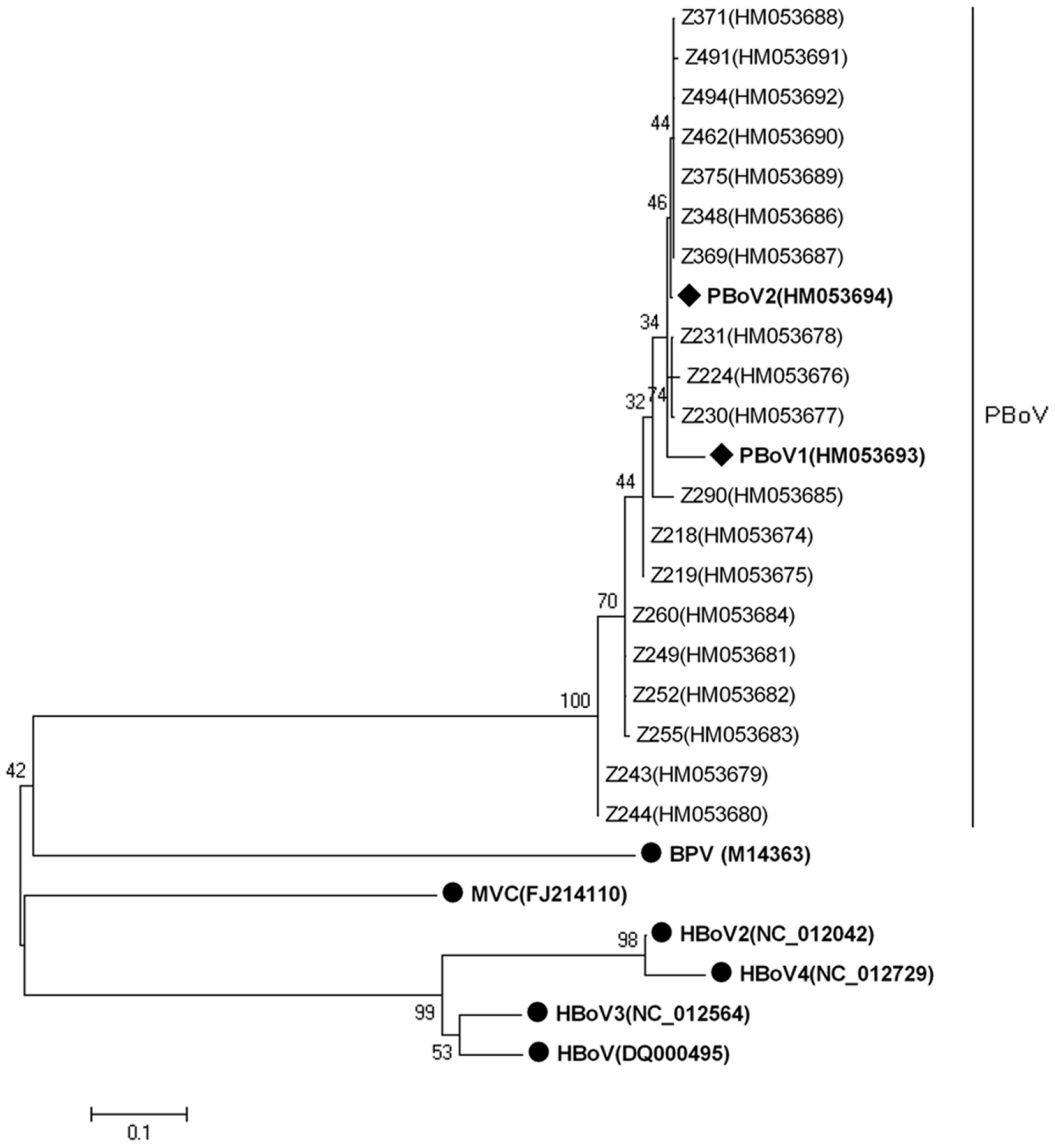
Phylogenetic analyses of partial *NS1* gene nucleotide sequences from PBoV and other bocavirus members. The phylogenetic tree was constructed by the neighbor-joining method with 1,000 bootstrap replicates, using the MEGA 4.1 software. Black circles designate reference strains, and the others are sequences generated from the present study. PBoV1 and PBoV2 are indicated with black diamonds. MVC: minute virus of canines; BPV: bovine parvovirus. HBoV: human bocavirus.

Two primers were designed based on sequence A to amplify a partial *NS1* gene of PBoV in 397 stool samples from healthy piglets. Fifty (12.59%) samples were identified as positive for PBoV; most of them were collected in October and November 2006. These *NS1* gene sequences displayed 94.3–100% identity with each other. The phylogenetic tree was constructed by the neighbor-joining method with 1,000 bootstrap replicates, using the MEGA 4.1 software, and revealed that all of these sequences were grouped together in one cluster, but separate from other bocaviruses (Figure 1).

### Genomic analysis of porcine bocavirus

According to sequences A and B, specific primer PCR and genome walking PCR were used to amplify the complete sequences of PBoV. Two nearly full-length genomic sequences except the termini were obtained: PBoV1 (5,173 nt; Acc. No. HM053693) and PBoV2 (5,186 nt; Acc. No. HM053694). The base composition of PBoV1 and PBoV2 was 27.4–28% A, 25.0–25.1% C, 29.4–29.9% G, and 17.5–17.7% T, with 54.4–54.9% GC. The two nearly full-length sequences showed the highest identity to MVC at the nucleotide level in the BLAST search, using default parameters.

**Figure 2:**
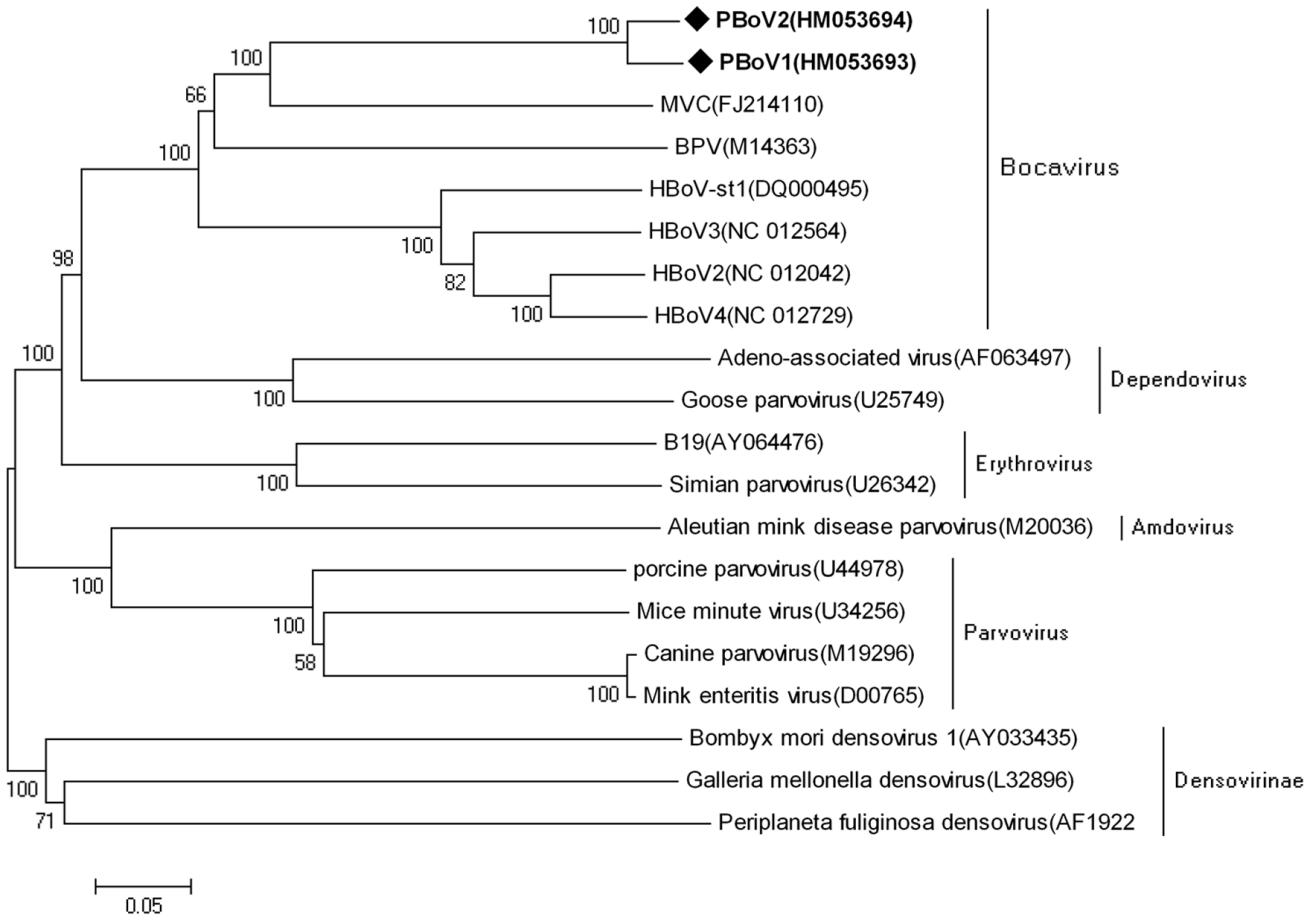
The phylogenetic analyses of viruses in the *Parvoviridae* family. The phylogenetic tree was constructed from nearly full-length nucleotide sequences of subfamilies *Parvovirinae* (genera *Parvovirus*, *Erythrovirus*, *Dependovirus*, *Amdovirus*, and *Bocavirus*) and *Densovirinae*, using the MEGA 4.1 software (neighbor-joining method with 1,000 bootstrap replicates). PBoV1 and PVoB2 are labeled with black diamonds. MVC: minute virus of canines; BPV: bovine parvovirus; HBoV: human bocavirus; Pbo-likeV: porcine boca-like virus.

Phylogenetic tree was constructed based on the alignments of PBoV1, PBoV2 and other viruses in the *Parvoviridae* family, the results indicated that PBoV was grouped with other bocaviruses (HBoV, MVC and BPV) but formed a distinct cluster (Figure 2).

**Figure 3:**
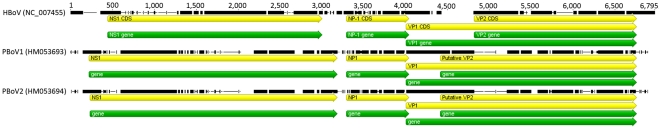
Map of the genomic organization of PBoV and HBoV. The map was constructed using the Geneious 4.8.5 software. Genus *bocavirus* has a non-structural *NP1* protein encoded by an ORF in the middle of the genome, which is a unique structure present in bocaviruses, but not found in most other parvoviridae members.

Putative open reading frames (ORFs) of PBoV were predicted using NCBI's ORF Finder, and the map of the genomic organization of PBoV and HBoV was constructed using the Geneious 4.8.5 software (Figure 3). The genomic organization of PBoV was closest to other known bocavirus. Similar to other bocaviruses, PBoV had three major ORFs encoding two non-structural proteins (*NS1* and *NP1*) and two capsid proteins (*VP1* and *VP2*). *VP2* is a truncated version of the *VP1* protein with an N-terminal deletion of 138 amino acids (*VP1*-unique region). Similar to HBoV, the *NS1* and *NP1* gene of PBoV are separate, with a short length of overlapping sequence between the 3′ terminus of *NP1* and the 5′ terminus of the *VP1* gene. The genus *bocavirus* has a non-structural *NP1* protein encoded by an ORF in the middle of the genome, which is a unique structure present in bocaviruses, but not found in most other parvoviridae members.

Compared with the amino acid sequences of MVC (FJ214110), BPV (M14363), and HBoV (DQ000495), PBoV1 and PBoV2 show 45.0–45.2%, 36.8–37.4%, and 42.2–43.1% identity in the *NS1* gene, 45.1–48.9% 41.7–42.2%, and 45.2–47.4% identity in the *NP1* gene, and 56.5–56.8%, 45.9–47.0%, and 51.7–51.8% identity in the *VP1* gene, respectively. These results confirmed that the new virus belonged to the *Parvoviridae* family, subfamily *Parvovirinae*, genus *Bocavirus*, corresponding to the tBLASTx results.

Although they share a similar genomic organization, the two strains, PBoV1 and PBoV2, had different genomic sequences. At the nucleotide level, they shared 93.6% and 94.2% identity in the nearly full-length and *NS1* gene sequences. At the amino acid sequence level, PBoV1 and PBoV2 showed 93.3%, 87.8%, and 92.8% identity in the *NS1*, *NP1*, and *VP1* proteins. Additionally, when compared with PBoV1, PBoV2 has two and four extra amino acids in its *NP1* and *VP1* proteins, respectively.

### Additional bocavirus found in piglets

In the process of amplifying the complete PBoV sequences in various samples, two additional samples (6V, 7V) were positive for 2,407 bp and 2,434 bp amplification products, respectively, which included a partial *NP1* gene and complete *VP1* gene. Interestingly, these two sequences were not grouped with PBoV1 and PBoV2, and they only shared 53.9–55.6% DNA and 41.9–43.3% amino acid sequence similarities with them. When compared with the *VP1* amino acid sequences of other Bocaviruses, samples 6V and 7V showed 45.4–45.7% identity with MVC, 44.1–44.5% with BPV, and 43.1–43.3% with HBoV. Furthermore, 6V and 7V were only 92.2% and 92.4% similar to each other at the DNA and amino acid sequence levels, respectively.

### Comparison with Pbo-likeV

In 2009 in Sweden, Blomstrom *et al.* [24] reported the detection of a novel porcine boca-like virus (Pbo-likeV) with a 1,879-bp sequence, which included a complete *NP1* gene and partial *VP1/VP2* gene. Sequence analysis demonstrated that it was most closely related to HBoV, MVC, and BPV, sharing 33.1–35.6% identity with the *NP1* gene of these bocaviruses Compared with PBoV1 and PBoV2, Pbo-likeV shared only 47.3–49.2% identity in *NP1* protein sequence.

**Figure 4:**
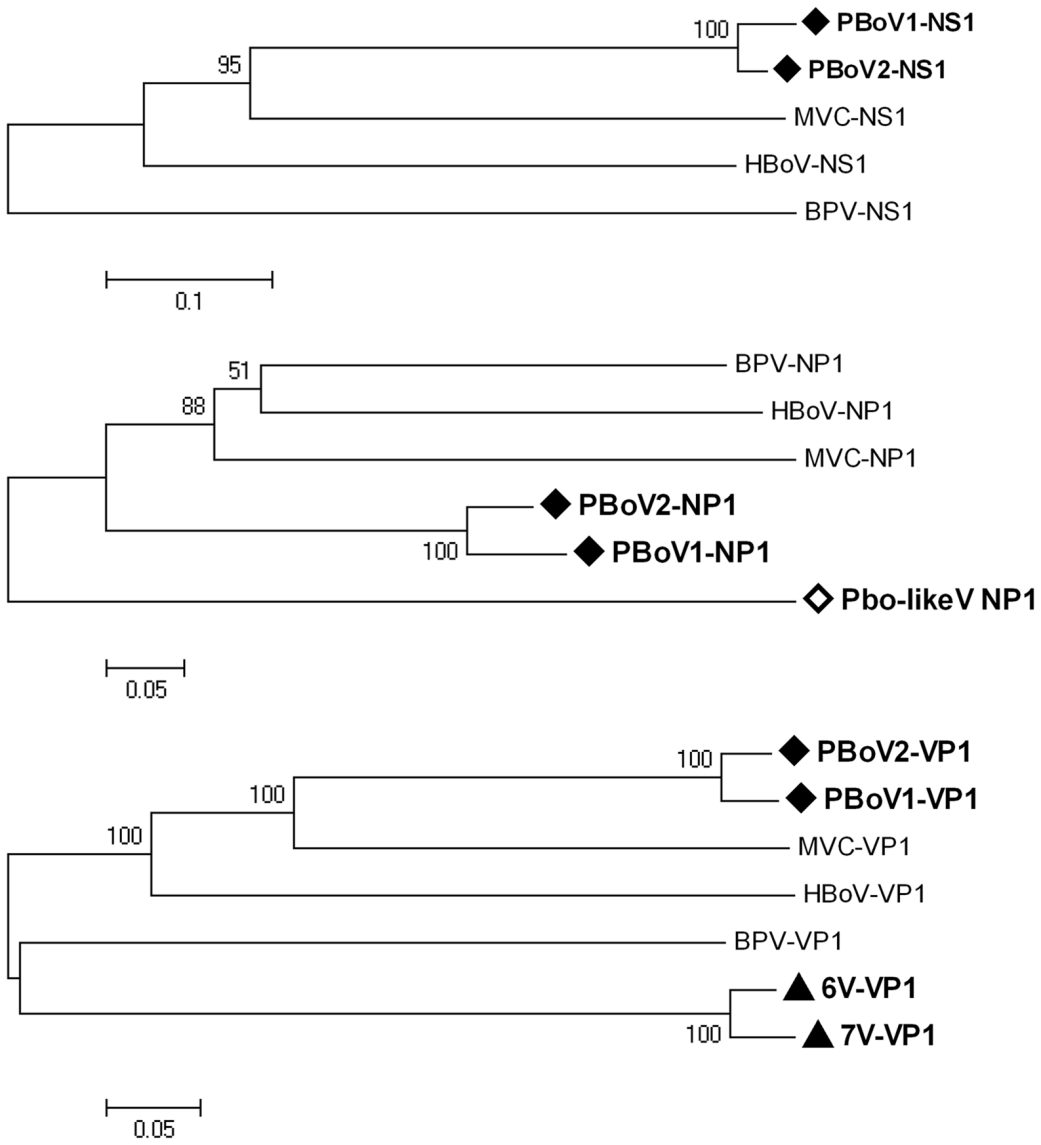
Phylogenetic relationships of three different protein (*NS1*, *NP1*, *VP1*) sequences in the *bocavirus* genus. The phylogenetic tree was constructed from amino acid sequences using the MEGA 4.1 software (neighbor-joining method with 1,000 bootstrap replicates). PBoV1 and PBoV2 are labeled with black diamonds, 6V and 7V are labeled with black triangles, and Pbo-likeV is labeled with an open diamond. MVC: minute virus of canines; BPV: bovine parvovirus; HBoV: human bocavirus; Pbo-likeV: porcine boca-like virus.

Phylogenetic trees of three different proteins (*NS1*, *NP1*, *VP1*) between PBoV and other members of the genus bocavirus were constructed from amino acid sequences, indicating that Pbo-likeV, PBoV1, PBoV2, 6V and 7V formed several separate clusters (Figure 4).

**Figure 5:**
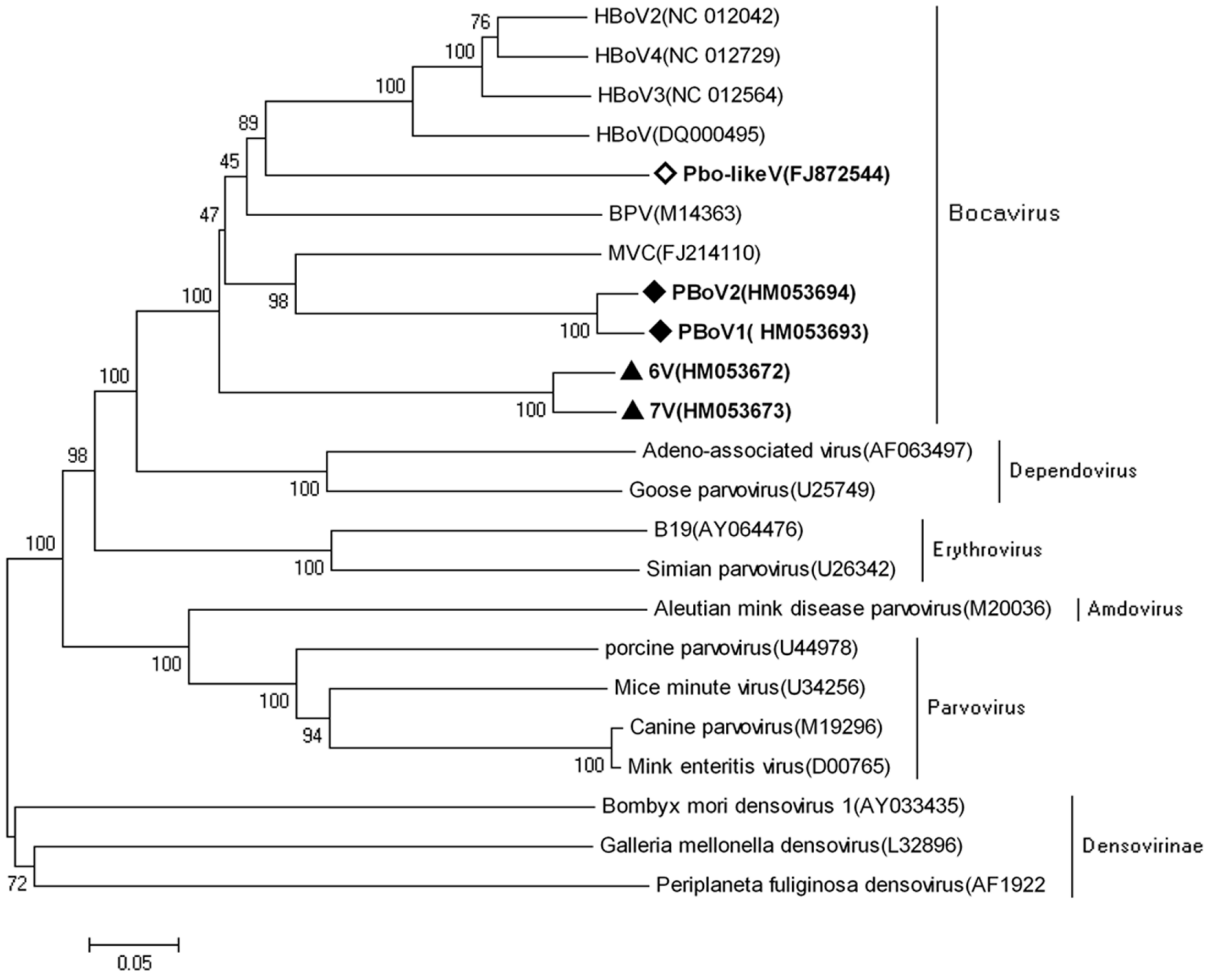
Phylogenetic relationships of the common region among PBoV, Pbo-likeV and other members of parvoviruses. The phylogenetic tree was constructed using the MEGA 4.1 software (neighbor-joining method with 1,000 bootstrap replicates). PBoV1 and PBoV2 are labeled with black diamonds, 6V and7V are labeled with black triangles, and Pbo-likeV is labeled with an open diamond. MVC: minute virus of canines; BPV: bovine parvovirus; HBoV: human bocavirus; Pbo-likeV: porcine boca-like virus.

Because the data for 6V, 7V, and Pbo-likeV were not complete sequences, we aligned them with other parvoviruses. By comparing only the regions in common from the middle of the *NP1* gene to the middle of the *VP1* gene (about 1,300–1,500 bp in length), Pbo-likeV showed 54.5–59.9% identity with PBoV1, PBoV2, 6V, 7V, and other bocaviruses (MVC, BPV, HBoV) in this region. Phylogenetic tree of this common region among these parvoviruses also indicated that Pbo-likeV, PBoV1, PBoV2, 6V and 7V formed three distinct clusters, but grouped in the *bocavirus* genus (Figure 5).

### Putative phospholipase A_2_ (PLA_2_) motif in bocaviruses and other parvoviruses

**Figure 6:**
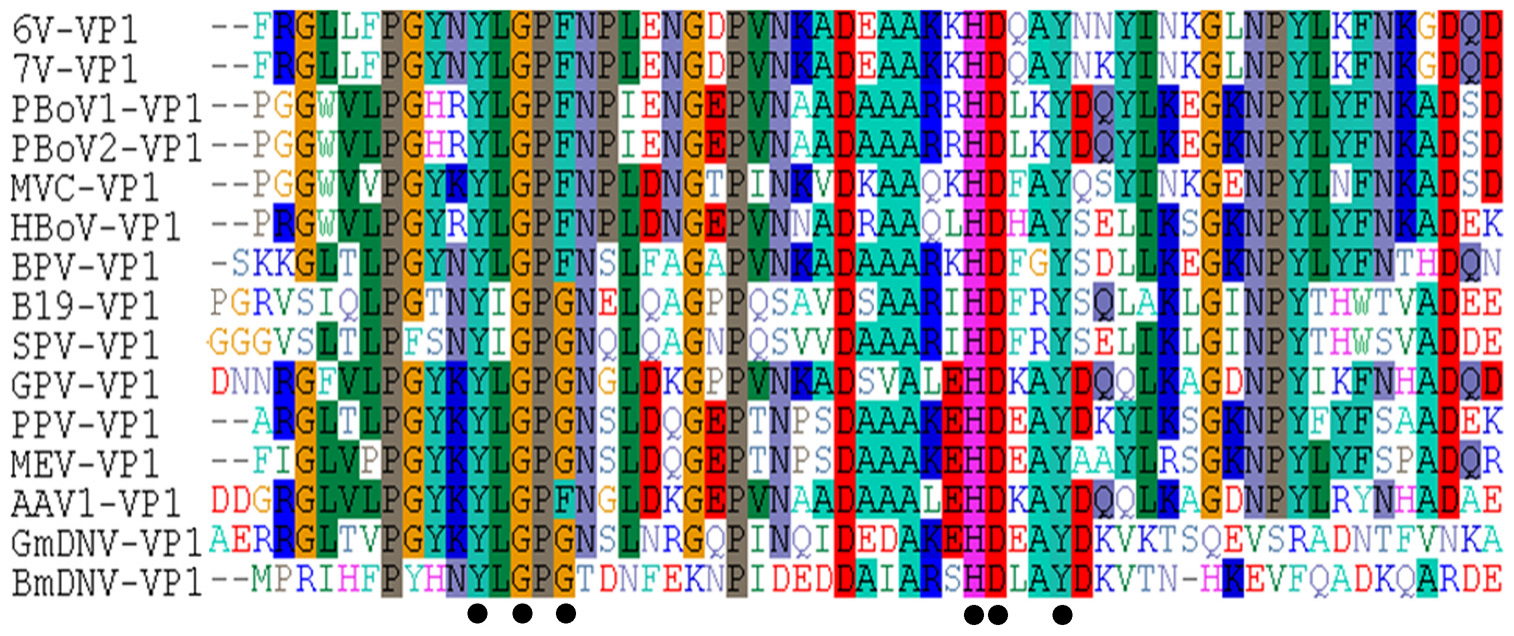
Putative phospholipase A_2_ (PLA_2_) motif in members of the genus *bocavirus* and other parvoviruses. It was constructed using BioEdit software. The HDXXY and YXGXG motifs of PLA_2_ are indicated by black circles at the bottom. The HDXXY motif is strongly conserved among parvoviruses, whereas the latter motif exists as YXGXF in bocaviruses, instead of the YXGXG motif found in most parvoviruses. MVC: minute virus of canines; BPV: bovine parvovirus; HBoV: human bocavirus; B19: human parvovirus B19; SPV: simian parvovirus; GPV: goose parvovirus; PPV: porcine parvovirus; MEV: mink enteritis virus; AAV1: adeno-associated virus-1; GmDNV: Galleria mellonella densovirus; BmDNV: Bombyx mori densovirus.

*VP1* protein sequences of bocaviruses and some other parvoviruses were aligned, and a conserved domain in the *VP1* unique protein (*VP1*U) was found (Figure 6). Similar to other parvoviruses, PBoV1, PBoV2, 6V, and 7V have a putative secretory phospholipase A_2_ (sPLA_2_) motif in the *VP1*U. In the alignment, a conserved HDXXY motif in the catalytic center of sPLA_2_ was found in the *VP1*U sequence of bocaviruses and other parvoviruses. Furthermore, the conserved Ca^2+^ binding loop of sPLA_2_ is an ‘YXGXF’ motif in bocaviruses, rather than the ‘YXGXG’ motif found in most parvoviruses, except AAV1.

### Recombination analysis between PBoV and other parvoviruses

Multiple alignments revealed that PBoV1 and PBoV2 share 28.7–55.6% and 30.2–56.8% DNA sequence identity with other members of the subfamily Parvovirinae, respectively. No recombination signal was found in either PBoV1 or PBoV2 with other bocaviruses by SimPlot analysis (data not shown).

## Discussion

In the current study, we identified a group of novel candidate porcine bocaviruses (PBoV) in the stools of piglets, the closest neighbors of which were MVC, BPV, and HBoV. The nearly full-length genomic sequences, except the termini, of two strains (PBoV1, PBoV2) were determined. Additionally, about 2,400-bp sequences was determined in two other strains (6V, 7V). Phylogenetic analyses revealed that these PBoVs were not closely related to other known porcine parvoviruses. Furthermore, they had a non-structural *NP1* protein encoded by an ORF in the middle of the genome, a unique structure found in bocaviruses, but not in most other parvoviridae members. The *NP1* protein is a highly phosphorylated protein, although its function has not yet been determined [4].

The PBoVs grouped with other bocaviruses (HBoV, MVC, BPV) in phylogenetic trees. However, they share only about 37.4–45.0% identity with other bocaviruses in the *NS1* gene, confirming them as a new member in the *bocavirus* genus, *parvovirinae* subfamily, *parvoviridae* family. PBoV1 and PBoV2 only share 94.2% identity in their *NS1* gene sequences at the nucleotide level. Species with non-structural gene genetic homology of less than 95% are defined as a new species in the *bocavirus* genus, in accordance with the ICTVb criteria (http://www.ictvdb.org/Ictv/fs_parvo.htm). Thus, these two strains can be defined as two new species of PBoV.

Pbo-likeV was detected in 2009 by Blomstrom *et al.* in the background of porcine circovirus type 2-induced post-weaning multi-systemic wasting syndrome (PMWS). They proposed that it was a novel porcine parvovirus with closest relationship to bocaviruses. But they only obtained a 1,879-bp sequence with a complete *NP1* gene and partial *VP1/VP2* gene [24]. Subsequently in China, Zhai *et al.* [25] detected this virus based on the VP1/2 gene of the Swedish Pbo-likeV strain, and the expected product size was 496-bp. Alignment results showed that these fragments have 99–100% identity with the Swedish Pbo-likeV strain. It was reasonable that the virus they detected should be named Pbo-likeV strains in China instead of porcine bocavirus (PBoV).

Although sequence analyses suggested PBoV1-2, 6V-7V and Pbo-likeV all belong to the *bocavirus* genus, they showed marked variation in their *NS1*, *NP1* and *VP1/VP2* sequences, and phylogenetic analyses also indicated they were in three separate clusters, suggesting the likelihood of there being more than one new porcine bocavirus species in this genus. However, complete sequences for Pbo-likeV and 6V-7V were not obtained, and a new species is defined by non-structural (*NS1*) gene homology, so whether these are new bocavirus species is, as yet, unclear.

Previous studies have discovered conserved sequences of about 40 amino acids in the N terminal region of the *VP1* protein in most parvoviruses, whereas the remaining sequences of *VP1* vary greatly; this conserved domain has been demonstrated to have sPLA_2_-like activity [26]–[29]. This activity has been suggested to be a key for the efficient transfer of the viral genome from late endosomes/lysomes to the nucleus to initiate viral replication in parvoviruses, and amino acid substitution in the active site of the sPLA_2_ motif would inactivate enzymatic activity, eliminating viral infectivity [26], [28], [30]–[32]. A conserved HDXXY motif in the catalytic center of sPLA_2_, and the YXGXF motif, similar to the YXGXG motif in the conserved Ca^2+^-binding loop of sPLA_2_, were also found in the *VP1*U sequences of PBoV1-2 and 6V-7V. Although the conserved Ca^2+^-binding loop of sPLA_2_ in all bocaviruses contains YXGXF, rather than the YXGXG motif found in most other parvoviruses, we have demonstrated previously that HBoV with an YXGXF motif also possesses sPLA_2_ activity [31]. This suggests that the alternate motif found in PBoV may also have sPLA_2_ activity, although further study is needed.

Previous studies have suggested the occurrence of recombination phenomena within parvoviral species [12], [13], [33]–[35]. In the current study, no recombination signal was found for either PBoV1 or PBoV2 with other bocaviruses, indicating that PBoV is not a recombination of other parvoviruses, but rather a novel member of the genus bocavirus. In addition to obtaining more PBoV genome sequences in the future, further study is needed to address exact phylogenetic relationships between various porcine bocavirus species and strains.

The severity of disease caused by parvoviruses varies by virus [34], [36]. Some parvoviruses cause mild disease or asymptomatic infections [37], [38], whereas others can result in fetal death and abortion in animals [34], [39], [40]. The members of genus bocavirus have a close relationship with respiratory and enteric diseases, especially in younger children and animals [4], [7], [11]–[16]. In the further study by Blomstrom *et al* in 2010, they detected Pbo-likeV in pigs with and without PMWS with infection rates of 88% and 46%, respectively[41], indicating that Pbo-likeV may have relationship with PMWS. Zhai's study[25] showed that the positive rates of Pbo-likeV (38.7%, 74/191) in pigs suffered from respiratory tract symptoms were significantly higher than those (7.3%, 3/41) in healthy pigs, so they proposed that this virus might be an emerging virus for swine respiratory tract diseases. The PBoVs found in the current study had a high prevalence (12.59%) in stool samples of piglets, suggesting that swine is a host for PBoV, and that they may multiply in the intestinal tract of piglets, as do other bocaviruses. With the inclusion of only healthy piglets and the absence of serologic and cytology data, it is not yet known whether PBoV can cause gastroenteritis or other diseases in swine. We are conducting a case control study to clarify the role of PBoV in gastroenteritis in piglets.

In the current study, to the best of our knowledge, several different bocaviruses with two nearly full-length genome sequences were identified for the first time in porcine samples and these are proposed to be new bocavirus members in pigs. Our findings indicate that many new potential bocavirus species exist in pigs. Further study is needed to explore their exact roles, including the clinical significance and epidemiology of their infection, and the precise evolutionary relationships among them. Although MVC, BPV, and human bocaviruses were discovered in recent decades, the biological characteristics of the genus of *bocavirus*, such as virus replication and assembly, and relatedness to diseases are still not fully understood. The observation of PBoV and other potential new members of the *bocavirus* genus can perhaps help in the molecular and functional characterization of bocaviruses in the near future.
